# Mapping delays in breast cancer care during COVID-19: Lessons from the Brazilian Public Health System (SUS)

**DOI:** 10.1016/j.clinsp.2025.100696

**Published:** 2025-05-23

**Authors:** Diego Wallace Nascimento, José Roberto Filassi, Rodrigo Gonçalves, Edmund Chada Baracat, José Maria Soares Júnior, Bruna Salani Mota

**Affiliations:** Mastology Division, Department of Gynecology and Obstetrics, Faculdade de Medicina da Universidade de São Paulo, São Paulo, SP, Brazil

In March 2020, the World Health Organization (WHO) classified COVID-19 as a global pandemic, leading health systems around the world to shift financial and structural resources to curb the virus's spread.^1^ In Brazil, the Unified Health System swiftly reorganized to meet the demands of COVID-19, leading to significant disruptions to public health policies related to cancer screening,^2^ treatment, and follow-up, particularly for breast cancer.

These disruptions have had a direct impact on patient outcomes, contributing to an increase in the diagnosis of advanced-stage breast cancer, as reported by various health organizations.^3^, ^4^, ^5^, ^6^ Delays in screening, treatment, and follow-up are expected to negatively affect long-term outcomes, including overall and disease-free survival, while also increasing the cost of care for the public healthcare system.

Despite the growing need for timely interventions, Brazil's oncology data systems present substantial limitations in tracking the patient journey from initial screening to definitive treatment within the SUS. The lack of integrated and comprehensive data hinders strategic planning and impedes the development of effective emergency policy responses.

To address this gap, the Ministry of Health developed PAINEL-Oncology, a data management tool integrated with DATASUS.^7^^,^^8^ This tool links multiple information systems within the SUS, including:

• SIA (Outpatient Information System).
• BPA-I (Individualized Outpatient Production Bulletin).
• APAC (Authorization for High-Complexity Procedures).
• SIH (Hospital Information System).

These databases are linked to the National Health Card (CNS) and the ICD-10 codes. Since 2019, the PAINEL-Oncology platform has integrated retrospective data from the Cancer Information System – Breast and Cervical Cancer (SISCAN), covering records dating back to 2013.

The integration enabled by PAINEL facilitates nationwide surveillance of breast cancer cases and allows for the evaluation of the time interval between diagnosis and initiation of treatment. Treatment start times are categorized as ≤ 30 days, 31–60 days, and > 60 days (Table 1). Unfortunately, a substantial proportion of patients commence therapy more than 60 days after diagnosis, underscoring the systemic delays prevalent across many locations.

**Table 1:** Time in days by Brazilian region to initiate treatment from the date of diagnosis in patients with malignant breast neoplasms and ductal carcinoma in situ.

Region diagnosis	Up to 30 days	31‒60 days	More than 60 days	No treatment information	Total
**Year**				**2018**	
Total	11.275	7.790	21.855	5.329	46.249
North Region	418	277	904	178	1.777
Northeast Region	2.642	1.969	5.106	1.595	11.312
Southeast Region	4.647	3.340	10.770	2.186	20.943
South Region	2.779	1.766	3.896	1.103	9.544
Midwest Region	789	438	1.179	267	2.673
**Year**			**2019**		
Total	12.065	8.574	24.154	11.173	55.966
North Region	482	342	933	644	2.401
Northeast Region	2.783	2.231	5.499	3.032	13.545
Southeast Region	5.168	3.735	11.861	4.877	25.641
South Region	2.868	1.833	4.431	2.291	11.424
Midwest Region	763	433	1.430	329	2.955
**Year**			**2020**		
Total	10.538	9.002	20.620	11.138	51.298
North Region	422	302	1.021	481	2.226
Northeast Region	2.328	2.240	4.955	2.816	12.339
Southeast Region	4.584	4.054	9.558	5.045	23.241
South Region	2.638	1.941	3.713	2.313	10.605
Midwest Region	566	465	1.373	483	2.887
**Year**			**2021**		
Total	10.529	9.753	25.011	13.167	58.460
North Region	405	341	1.299	298	2.343
Northeast Region	2.344	2.565	6.143	4.011	15.063
Southeast Region	4.550	4.332	11.560	5.999	26.441
South Region	2.683	1.959	4.415	2.403	11.460
Midwest Region	547	556	1.594	456	3.153

From 2018 to 2021, the numbers of patients diagnosed with malignant breast neoplasms or ductal carcinoma in situ within the SUS were 46,249; 55,966; 51,298, and 58,460 respectively (Fig. 1). The majority of cases were clustered in the southeastern region, presumably because of its larger population, stronger medical workforce, and better-developed infrastructure. In contrast, the North area recorded the lowest number of cases, indicating demographic and structural differences. In 2020, the first year of the pandemic, there was a significant reduction in diagnoses compared to those in 2019.

**Fig. 1:**
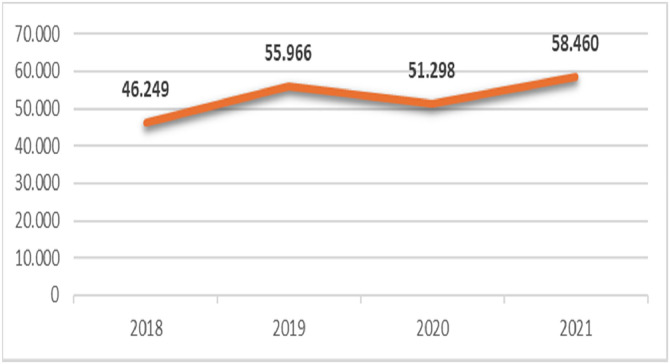
Number of new cases of malignant breast neoplasms and ductal carcinoma in situ in Brazil from 2018 to 2021.

The COVID-19 pandemic has had a profound global impact on healthcare systems. The sudden surge in demand for clinical treatment and hospital admissions during the pandemic's acute phase placed an extraordinary burden on healthcare professionals and available resources. To prevent a systemic collapse, many countries were forced to scale back or interrupt essential health services ‒ including routine diagnostics, screening programs, and elective procedures ‒ for chronic diseases, infectious conditions, and certain neoplasms. As a result, prevention and timely treatment of these conditions were compromised, leading to a rise in complications and increased mortality from otherwise preventable illnesses.

Especially and breast cancer care, the COVID-19 outbreak has exposed significant deficiencies in Brazil's infrastructure. Utilizing tools like PAINEL-Oncology to map delays provides critical insights and highlights the critical necessity to reform cancer treatment pathways. Ensuring timely access to diagnosis and treatment within the SUS must be prioritized, not only during public health emergencies but also as a sustained commitment to improving health outcomes.

## Data access statement

Brazilian Ministry of Health.

## Funding

This work received no specific grant from any funding agency in the public, commercial, or non-profit sectors.

## CRediT authorship contribution statement

**Diego Wallace Nascimento:** Conceptualization, Data curation, Investigation, Writing – review & editing. **José Roberto Filassi:** Conceptualization, Supervision. **Rodrigo Gonçalves:** Conceptualization, Supervision. **Edmund Chada Baracat:** Conceptualization, Supervision. **José Maria Soares Júnior:** Conceptualization, Supervision. **Bruna Salani Mota:** Conceptualization, Data curation, Writing – review & editing.

## Declaration of competing interest

DWN ‒ Speaker Bureau Novartis and ATLS instructor.

RG – is currently employed by AstraZeneca R&D, but in a role not related in any way to the work presented in this manuscript.

The other authors declare that they have no affiliations with or involvement in any organization or entity with any financial interest in the subject matter or materials discussed in this manuscript.
